# Assessment of ITS2 Region Relevance for Taxa Discrimination and Phylogenetic Inference among Pinaceae

**DOI:** 10.3390/plants11081078

**Published:** 2022-04-15

**Authors:** Joanna Sokołowska, Hanna Fuchs, Konrad Celiński

**Affiliations:** 1 Department of Genetics, Institute of Experimental Biology, Faculty of Biology, School of Natural Sciences, Adam Mickiewicz University, Poznań, Uniwersytetu Poznańskiego 6, 61-614 Poznań, Poland; joanna.sokolowska@amu.edu.pl; 2 Institute of Dendrology, Polish Academy of Sciences, Parkowa 5, 62-035 Kórnik, Poland; hkijak@man.poznan.pl

**Keywords:** Pinaceae, internal transcribed spacer, ITS2, DNA barcoding, taxa identification

## Abstract

The internal transcribed spacer 2 (ITS2) is one of the best-known universal DNA barcode regions. This short nuclear region is commonly used not only to discriminate taxa, but also to reconstruct phylogenetic relationships. However, the efficiency of using ITS2 in these applications depends on many factors, including the family under study. Pinaceae represents the largest family of extant gymnosperms, with many species of great ecological, economic, and medical importance. Moreover, many members of this family are representatives of rare, protected, or endangered species. A simple method for the identification of Pinaceae species based on DNA is necessary for their effective protection, authentication of products containing Pinaceae representatives, or phylogenetic inference. In this study, for the first time, we conducted a comprehensive study summarizing the legitimacy of using the ITS2 region for these purposes. A total of 368 sequences representing 71 closely and distantly related taxa of the seven genera and three subfamilies of Pinaceae were characterized for genetic variability and divergence. Intra- and interspecies distances of ITS2 sequences as well as rates of sequence identification and taxa discrimination among Pinaceae at various taxonomic levels, i.e., the species complex, genus, subfamily, and family, were also determined. Our study provides a critical assessment of the suitability of the ITS2 nuclear DNA region for taxa discrimination among Pinaceae. The obtained results clearly show that its usefulness for this purpose is limited.

## 1. Introduction

The Pinaceae family is the largest extant family of all gymnosperms [1]. Within this family, 225 species have been distinguished, which are grouped in three subfamilies (Pinoideae Pilg., Laricoideae Melchior et Werdermann, Abietoideae Pilg.) and eleven genera, i.e., *Abies* Mill., *Cathaya* Chun & Kuang, *Cedrus* Belon ex Trew, *Keteleeria* Carriere., *Larix* Mill., *Nothotsuga* Hu ex C. N. Page, *Picea* A. Dietr., *Pinus* L., *Pseudolarix* Gordon, *Pseudotsuga* Carriere, and *Tsuga* Carriere [2]. Representatives of the Pinaceae are an extremely important component of temperate, boreal, subalpine, and subtropical forests in the northern hemisphere. Moreover, conifers from the Pinaceae family are of great economic importance, especially the genus *Pinus*, which has been used for many years as a valuable source of wood, resins, essential oils, and seeds [3]. Many studies also show that members of this family represent a valuable source of active substances with medicinal properties [4,5,6,7,8].

The high economic and medical value of members of this family may be a strong temptation to over-exploit particular species, obtain them illegally, or even falsify products containing them. Since many species of the Pinaceae family are rare, endangered, or protected, this can pose a serious threat. Another equally serious risk is the misidentification of certain species. There are many closely related taxa in the Pinaceae family with a very similar needle or cone morphology. Moreover, they have the same ecological niches, similar geographic ranges, and often grow together in sympatric populations. In such populations, gene flow occurs, which leads to the formation of hybrid individuals with a phenotype intermediate to the parent taxa. Thus, the processes of hybridization and introgression additionally hinder the simple and effective assignment of such individuals to particular species. One of the best examples of such a complex system involving all of the above-mentioned factors is the European mountain pine association, *Pinus mugo* Turra *sensu lato*. This complex has been the subject of research using various techniques and methods for several decades [9,10,11,12,13,14,15]. However, the main problem in this complex remains the same, i.e., finding easy-to-use specific diagnostic determinants for individual taxa, preferably based on DNA markers.

The unambiguous identification of plant material or distinguishing taxa plays an important role not only in the authentication of Pinaceae-containing products, but also in establishing genetic relationships in this family, characterizing the genetic resources of its taxa, determining their geographical distribution, developing conservation plans for individual taxa, monitoring gene flow, and finally analyzing various evolutionary processes, such as hybridization or introgression.

DNA barcoding is a popular method of species identification using short and standardized gene fragments as molecular markers [16]. This rapid approach has commonly been used in taxonomic research for species-level identification in different domains of the tree of life for several years. The undeniable features of good DNA barcode regions are easy PCR amplification and sequencing, and most importantly, ensuring a high level of species discrimination [17]. The short length of the PCR amplicons facilitates work with very degraded DNA, e.g., from herbarium specimens. However, the effectiveness of identifying the analyzed biological material with DNA barcodes and assigning it to a specific family, genus, or species depends on many factors (including, among others, the analyzed family, DNA region or abundance of the reference base) and is not always satisfactorily high [18,19,20,21]. Therefore, in recent years, many studies have been carried out to circumvent these limitations by selecting appropriate regions of DNA that guarantee a high percentage of satisfactory identifications, both for universal barcoding and for species identification within individual families or genera [22,23,24,25,26]. One of the best known and widely used universal DNA barcode regions is the internal transcribed spacer 2 (ITS2) [27]. This short nuclear region, due to its properties, is also used to reconstruct phylogenetic relationships at the species and genus level [28,29,30,31,32]. So far, ITS2 has been used to analyze taxa from the Pinaceae family rather sporadically, while evaluating the usefulness of this region as barcode DNA for various groups of plants and animals [26,27].To the best of the authors’ knowledge, so far, there is no comprehensive study summarizing the validity of using the ITS2 region for the discrimination and identification of taxa as well as phylogenetic inference in this family carried out on a large number of species and sequences.

Therefore, the main objective of this study is to determine whether ITS2: (1) is a useful DNA marker for the discrimination and identification of both distant and closely related taxa in the Pinaceae family; (2) has a sufficiently high potential for the authentication of products containing Pinaceae representatives; and (3) is valuable nuclear region for inferring phylogenetic relationships of conifers in this family.

## 2. Results

### 2.1. Genetic Variation of ITS2 Sequences in Pinaceae

A total of 368 sequences representing 71 Pinaceae conifer taxa were analyzed (Table S1), of which 346 sequences were downloaded from GenBank and the remaining 22 sequences were obtained in this study and deposited in the same genetic database (Table S2). The accession numbers of the newly obtained sequences are shown in Table S3.

The genetic variability of the ITS2 sequence in the Pinaceae family was precisely characterized. All analyses and calculations were performed separately for four different taxonomic levels, i.e., species complex: *Pinus mugo* complex; genus: *Abies*, *Keteleeria*, *Tsuga*, *Larix*, *Pseudotsuga*, *Picea,* and *Pinus*; subfamily: Abietoideae, Laricoideae, Pinoideae, and family: Pinaceae.

The obtained results indicate that the analyzed ITS2 sequences in Pinaceae differ significantly in the values of the basic parameters describing genetic variation depending on the considered taxonomic level (Table 1). At the species complex level, represented by the closely related European mountain pine complex, *Pinus mugo* complex (PMC), no sequence variation was observed. At the genera level, the alignment length varied only slightly. The shortest alignment length was noticed for the genus *Pseudotsuga* (232 bp), while the longest for the genus *Pinus* (252 bp). The number of conserved sites (CS) ranged from 70 to 237. The highest percentage of calculated conserved sites (CS) was observed for the genus *Pseudotsuga* (100%) and the lowest for the *Pinus* genus (27.78%). The number of variable sites (VS) ranged from 0 (for the genus *Pseudotsuga*) to 177 (for the *Pinus* genus), which gives 0% and 70.24% respectively. The genus *Pseudotsuga* was also characterized by zero parsimony informative sites (PIS) and singleton sites (SS), and consequently also zero overall mean distance (OMD). On the other hand, the highest values of these coefficients were recorded for the genus *Pinus* (28.97%) and *Keteleeria* (48.5%), for PIS and SS, respectively. The highest OMD value (0.236) was found for the genus *Keteleeria*. At the subfamily level, the alignment length varied from 233 bp to 254 bp. The number of conserved sites ranged from 61 to 148. The highest percentage of calculated conserved sites (CS) was observed for the Laricoideae (63.52%) and the lowest for the Pinoideae subfamily (24.21%). The number of variable sites varied substantially from 85 to 187 within the subfamilies. The lowest proportion of variable sites (VS) was observed for the Laricoideae (36.48%) and the highest for the Pinoideae (74.21%) followed by Abietoideae (73.23%). The number of parsimony informative sites (PIS) ranged from 48 (Laricoideae) to 107 (Pinoideae), which is 20.60% and 42.4%, respectively. The values of the number of singleton sites (SS) and overall mean distance (OMD) were also the lowest for the members of the Laricoideae subfamily and amounted to 15.88% and 0.023, respectively. The highest percentage of calculated singleton sites was observed for the Abietoideae subfamily (38.19%), and the highest overall mean distance (OMD) was noticed for the Pinoideae (0.149). At the family level, the estimated alignment length was equal to 270 bp. The proportion of calculated conserved sites (CS), variable sites (VS), parsimony informative sites (PIS), singleton sites (SS) was: 17 (6.30%), 250 (92.59%), 196 (72.5%), and 49 (18.15%), respectively.

The overall mean distance (OMD) for the Pinaceae is 0.342. In general, the highest values of the basic parameters characterizing the genetic variation of the ITS2 sequence in Pinaceae were found at the family level, and the lowest at the species complex level.

### 2.2. Genetic Divergence within and between Pinaceae Taxa

MEGA version X was used to calculate the genetic divergence of ITS2 sequences in the Pinaceae family. Table 2 summarizes in detail the values of five genetic divergence parameters, i.e., all interspecific distance, minimum interspecific distances, theta, all intraspecific distance, and coalescence depth obtained in this study at the species complex, genus, subfamily, and family levels.

At the *P. mugo* complex level, all genetic divergence indices were zero. At the genera level, the highest all interspecific and all intraspecific variation was observed in the genus *Keteleeria* (0.1998 ± 0.0973 and 0.1972 ± 0.1919, respectively). This genus was also characterized by the highest value of theta (0.1995 ± 0.0250) and coalescent depth (0.2958 ± 0.2874). The lowest values of the above-mentioned coefficients were observed for the genus *Picea*. The genus *Pseudotsuga* was the only one represented by only one taxon. Therefore, the value of all five coefficients for this type was zero. At the subfamily level, the lowest all interspecific and all intraspecific distances were revealed for Laricoideae (0.0436 ± 0.0083 and 0.0063 ± 0.0011). In turn, the highest all interspecific distance was found in Pinoideae (0.1702 ± 0.0059), and the highest all intraspecific distance in Abietoideae (0.0370 ± 0.0291). For all analyzed subfamilies, the minimum interspecific distance was equal to 0.0000. The theta value ranged from 0.0222 for Laricoideae to 0.1492 for Pinoideae. The highest value of coalescent depth was observed for Abietoideae (0.0562) while the lowest (0.0249) for Laricoideae. At the family level, all interspecific and all intraspecific distances parameters were 0.3586 and 0.0294, respectively. The theta value was equal 0.3417 and coalescent depth value was 0.0423.

**Figure 1:**
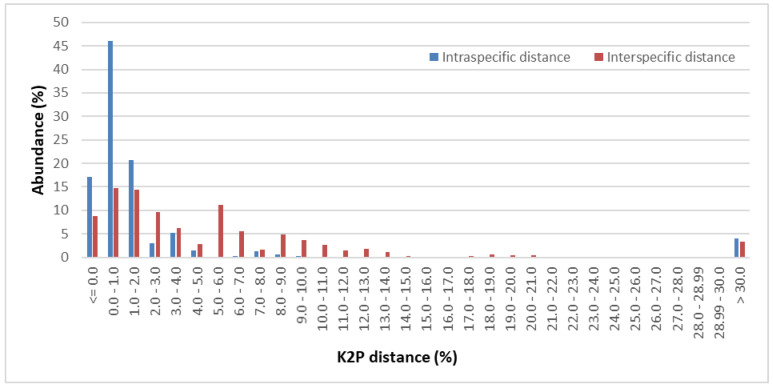
Abundance of all intraspecific and all interspecific K2P pairwise distance in Pinaceae.

The pairwise genetic distance-based method was used to determine the genetic divergence of the analyzed ITS2 sequences. Figure 1 shows the relative distribution of all intraspecific and all interspecific distances obtained for the analyzed samples from the Pinaceae family. The value of intraspecific distance in the range from 0.0% to 1.0% constituted 46.04% of the total observation, while the values of interspecific distance in the range of 0.0 to 1.0 and 1.0 to 2.0 were respectively 14.74% and 14.39%. Overall, the vast majority of intraspecific distance values (95.06%) are in the range from 0% to 8%, with 83.93% in the range from 0.0% to 2.0%. The interspecific value of the distance in the range from 0% to 14% constitutes 90.49% of the calculations, and the range of distance from 0% to 6% constitutes 67.75% (Table S4).

### 2.3. Rates of Sequences Identification and Taxa Discrimination among Pinaceae

BLAST1 and TAXONDNA/Species Identifier 1.8 were used to determine the percentage of correct, ambiguous, and incorrect ITS2 sequence identification and taxa discrimination among Pinaceae.

**Figure 2:**
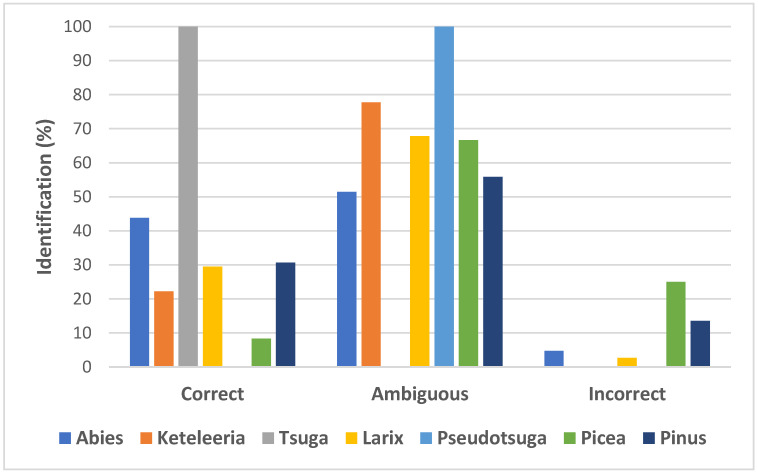
Species discrimination rates based on BLAST1 method.

Using the BLAST1 method, the correct ITS2 sequence identification rate was the highest for the *Tsuga* genus (100%) and the lowest for *Pseudotsuga* (0%). More than 40% of ITS2 sequences were correctly identified in the genus *Abies* and just over 30% in the genus *Pinus*. In contrast, in the genus *Picea*, only 8.33% of the ITS2 sequences were correctly identified. Ambiguous ITS2 sequences identification was highest for *Pseudotsuga* (100%) and lowest (0%) for *Tsuga*. Generally, for six out of seven analyzed Pinaceae genera, the value of ambiguous identification rate was more than 50%. Incorrect identification rates were highest for *Picea* (25%), followed by *Pinus* (13.51%) and *Abies* (4.76%). Overall success in correct species discrimination based on the BLAST1 method and ITS2 sequences was moderate (32.88%). Figure 2 and Table S5 summarize the results obtained with BLAST1 method.

TAXONDNA/Species Identifier 1.8 program, using “Best Match” (BM) and “Best Close Match” (BCM) options, was used to calculate the percentage of correct ITS2 sequence identification at the species complex, genus, subfamily, and family level for taxa represented in the analysis by at least two sequences. The summary of the success of taxa identification for analyzed genera among the Pinaceae family using the TaxonDNA method is shown in Table 3. At the species complex level, represented by the closely related European mountain pine complex, *Pinus mugo* complex (PMC), 100% of the analyzed ITS2 sequences were ambiguously identified using the BM and BCM options. None of the ITS2 sequence in this species complex were identified as correct (0%) or incorrect (0%). At the genera level, *Pseudotsuga* and *Tsuga* were the most successfully discriminated (100%), while *Keteleeria* had the lowest discriminatory success. At the subfamily level, the highest success of identification was noticed for Laricoideae (23.48%), and the lowest success for the subfamily Abietoideae (17.80%). At the family level, the success rate of species discrimination among the Pinaceae was relatively low. Only 71 of 368 sequences were correctly identified to species level (19.2%) based on best match (BM) analysis. It is worth emphasizing that the ambiguous identification was over three times higher than the correct identification (74.1%). Incorrect identification concerned 7.5% of the sequences. Similar results were obtained based on the best close match (BCM) analysis.

### 2.4. Phylogenetic Inference

Phylogenetic inference was performed in the RaxML v8.2.11 [33] using 371 ITS2 sequences and the maximum likelihood (ML) method. The resulting phylogenetic tree sorted all analyzed Pinaceae taxa into the appropriate genera according to the commonly accepted taxonomy, most with high bootstrap support. Although the ITS2 sequences allowed the assignment of taxa to the different Pinaceae genera, their distinction within these genera was not so obvious. These observations are fully consistent with the results obtained by us with BLAST1 and Taxon DNA in terms of the correct sequence identification and discrimination of Pinaceae taxa, where the species level also turned out to be the most critical.

The overall topology of the obtained phylogenetic ML tree based on ITS2 sequences is consistent with the commonly accepted division of Pinaceae and strongly supports the concept of monophyly of particular Pinaceae genera (Figure S1).

## 3. Discussion

The main aim of the article was to assess the suitability of the ITS2 region for the discrimination and identification of taxa in Pinaceae and phylogenetic inference in this large family of conifers. Detailed analysis of the genetic variation of the ITS2 sequence was performed for the first time in one comprehensive study. Particular attention has been paid to precisely characterize the level of genetic variation in the ITS2 sequence at four different taxonomic levels and to determine the success of sequence and taxa discrimination using both phylogenetically distant and closely related conifers.

In contrast to some reports in the literature [26,27] our observations clearly showed that although ITS2 is easy to analyze, it has severe limitations (mainly low level of genetic variability) and is not the best choice for identification, authentication, or phylogenetic inference in Pinaceae. The finding was quite unexpected as there are quite a few ITS2 sequences in the databases. Moreover, many studies show that ITS2 is a very good DNA barcode region [22,23,25,34,35]. According to China Plant BOL Group [36], the nuclear ribosomal DNA region, ITS, is characterized by a much better ability to discriminate species compared to plastid regions. Internal transcribed spacer (ITS1, 5.8S, ITS2) is characterized by a higher rate of evolution than the coding regions, is inherited biparentally, and shows a high level of divergence at the species level, which allows it to be used to identify even closely related species [37]. This is one of the reasons why the assessment of the ability of ITS2 to discriminate between closely related taxa in the Pinaceae family was one of the goals of our work.

In seed plants, the length of ITS varies usually from 500 to 700 bp [38], while in gymnosperms it is much longer, especially in Pinaceae (1500–3700 bp) [37,39], except that half the length of ITS1 consists of subrepeats [40,41,42,43], which results in some difficulty in PCR amplification and typical Sanger sequencing reads. Hence, the availability of complete ITS sequences in genetic databases for different species is still very limited. Therefore, China Plant BOL Group has suggested using only the ITS2 sequence as an alternative to the complete ITS region, due to the corresponding variability of the primary and secondary structure [23,27,44].

There are many reports in the literature on the assessment of the identification potential of various genomic regions postulated for distinguishing and analyzing different groups of organisms that would be ideal DNA barcodes. For animals, the cytochrome c oxidase 1 subunit 1 has been proposed as the main bar code [16]. In the case of plant identification, this region will not work well due to the insufficient rate of nucleotide substitution in the plant’s mitochondrial genome [45]. To solve this problem, several promising highly variable plastid DNA regions have been proposed, including both coding and non-coding loci, which can be used singly or together in various combinations. In this way, among others, *rbcL* [45,46,47], *matK* [48,49,50,51,52], combination *rbcL + matK* [53], intergenic spacer region- *trnH-psbA* [46,49,50,54,55,56,57], *rpoB* [45,49,51], *rpoC1* [49,51], *atpF-atpH* [45], *ndhJ*, *ycf*5, or *accD* [51], were selected as valuable. Recently, the *ycf*1 region was indicated as very promising due to its very high variability, and even recommended as the main barcode in terrestrial plants [58]. Although the usefulness of *ycf*1 as a species-diagnostic marker has been demonstrated in the case of the Pinaceae family [59], it should be used in the analyses with caution as it was confirmed that this region is under positive selection in all *Pinus* plastomes [60], as well as frequent hybridizations within the genus *Pinus*, and finally the complex model of inheritance of the chloroplast genome [61]. Some difficulty in the widespread use of the complete *ycf*1 region may also arise from its considerable length, which makes it difficult to apply conventional Sanger sequencing with readings of 600–800 bp.

In this respect, nuclear markers, especially short ITS2, seem to be a better solution than chloroplast regions, especially given that the ITS2 region has so far been used quite successfully for the discrimination and identification of taxa in many angiosperm families. ITS2 turned out to be the best marker differentiating species from the Araliaceae family [24]. Several universal and popular DNA barcode regions (*matK*, *rbcL*, ITS2, *psbA-trnH*, and *ycf*5) were assessed by Liu [24] for their ability to identify 1113 sequences derived from 276 species from 23 genera. ITS2 correctly identified 85.23% and 97.29% of the sequences at the species and genus levels, respectively. Additionally, it was suggested that the *psbA-trnH* region could be an additional candidate DNA barcode for the identification of the Araliaceae family. Similarly, the high efficiency of the ITS2 region in discriminating taxa (>90% and 100% at the species and genus levels, respectively) was demonstrated in the Euphorbiaceae family based on the analysis of 1183 samples representing 871 species and 66 genera [34]. Similar results were obtained in the study of the Rutaceae family [62], where ITS2 was shown to be superior to the other six barcodes (*psbA-trnH*, *matK*, *ycf*5, *rpo*C1, *rbcL*, ITS). It was characterized by the highest interspecific divergence with regard to intraspecific divergence. Moreover, it has also been proven to be effective in distinguishing between closely related species. However, in our research, the effectiveness of the ITS2 sequence is zero at the *P. mugo* complex level. Our research clearly shows that the higher the taxonomic level, the higher the percentage of success in discriminating Pinaceae taxa. However, it seems that this percentage is decreasing not only with decreasing phylogenetic distance of analyzed samples, but also with increasing number of available sequences and individuals in the database.

ITS2 was also used with greater or lesser success in the analysis of the Apocynaceae family [22] or Asteraceae [35]. In the latter family, ITS2 was considered as a suitable, but not ideal, barcode for identifying species of high medical importance, belonging to the largest family of flowering plants. Compared to other barcodes, ITS2 was characterized by the greatest universality, specific divergence, and discrimination, which makes it a promising marker for Asteraceae authentication. In the Fabaceae family, ITS2 has been shown to be effective in identifying medicinal plants. It is worth noting that ITS2 also turned out to be an appropriate phylogenetic marker, but it did not solve all taxonomic problems [23]. This suggests that effective DNA barcoding using ITS2 varies from family to family and can be unreliable in complex taxonomic groups. In the Brassicaceae and Roasaceae families, ITS2 proved to be an imperfect barcode due to its low resolution [63,64].

As generally accepted, the ideal DNA barcode region should have a higher interspecies than intraspecies variation. In the case of the current study based on the pairwise genetic distance-based method (PWG-distance) for 368 ITS2 sequences from the Pinaceae family, the estimated inter-specific divergence parameter is higher than the intra-specific distances for all analyzed groups except for the *Pinus* genus. The DNA sequence similarity-based method, on the other hand, showed that in the case of the Pinaceae family, the success of taxa discrimination using ITS2 was relatively low. This shows that internal transcribed spacer 2 has some problems in identifying both subspecies and varieties, so closely related species are not properly distinguished.

Our results are fully consistent with those obtained for other gymnosperm families, including Podocarpaceae, the second largest family among gymnosperms, in which ITS2 was not characterized by too high an index of taxa discrimination [20]. Moreover, Yao`s [26] studies on several families of gymnosperms showed that ITS 2 had the least discriminatory success at the species level in comparison to other plant groups (mosses, ferns, monocotyledons, dicotyledons) as well as animals.

## 4. Materials and Methods

### 4.1. Sampling and Plant Materials

In our study, a total of 371 ITS2 sequences were analyzed, of which 368 sequences belonged to 71 Pinaceae taxa of 7 genera, and three *Podocarpus* sp. Sequences were outgroups only for phylogenetic analyzes (Tables S1 and S2). Of the 368 ITS2 Pinaceae sequences, 346 samples were downloaded from GenBank, and 22 sequences were obtained in this study by analyzing 20 individuals belonging to the *Pinus mugo* complex and 2 individuals of Scots pine (*Pinus sylvestris*) as the closest related taxa to the *Pinus mugo* complex. The analyzed specimens were collected in Poland, the Czech Republic and Germany (Table S3). The suitability of the ITS2 region to discriminate against taxa of the Pinaceae family was assessed by analyzing both phylogenetically distant and closely related taxa.

### 4.2. DNA Extraction and Next-Generation Sequencing

The 100 mg of fresh plant tissue was used to extract genomic DNA using the Genomic Mini AX Plant Spin kit (A&A Biotechnology, Gdańsk, Poland). The quality and concentration of the extracted DNA were verified using gel electrophoresis and a NanoDrop spectrophotometer (Thermo Fisher Scientific, Carlsbad, CA, United States). The genomic libraries were constructed with protocol: Ion Xpress™ Plus gDNA Fragment Library Preparation, using Ion Xpress Plus Fragment Library Kit (Pub. No. MAN0009847) (ThermoFisher Scientific, Waltham, MA, USA). Next-generation sequencing was performed on the GeneStudio™ S5 System (Thermo Fisher Scientific, Waltham, MA, USA) according to the manufacturer’s instructions using the protocol: Ion 540 ™ Kit—OT2 User Guide (Cat. No. A27753, Publication No. MAN0010850, Revision D). The reads generated from next-generation sequencing were assembled and annotated using the Geneious Prime 2020.2.5 package. Reads were mapped to MT735327 sequence from genbank using Genious mapper with default settings and minimum mapping quality 30%. Sequences that were mapped were subsequently assembled de novo using Geneious algorithm on default settings and annotated based on MT735327 sequence. The obtained 22 complete ITS2 sequences were submitted to GenBank. Each sequence was assigned an accession number (Table S2).

### 4.3. Data Validation

The sequences of internal transcribed spacer 2 from GenBank were downloaded using query “internal transcribed spacer 2 Pinaceae’’ (in July 2021). The nuclear ribosomal ITS2 sequences with ambiguous bases were discarded from further analyzes. In order to extract ITS2 sequence, the ITS2 database (Internal Transcribed Spacer 2 Ribosomal DNA Database) (version 3.0.13) available online (http://its2.bioapps.biozentrum.uni-wuerzburg.de (accessed on 20 August 2021) was used [65,66,67]. Single sequence species were excluded from the analysis.

### 4.4. Data Analysis

In order to carry out a deeper and more precise genetic characterization, i four taxonomic levels were considered, namely species complex, genus, subfamily, and family. ITS2 sequences were aligned using the ClustalW with default parameters available in MEGA version X [68]. Then, the length of the alignment was estimated and the percentage of conserved sites (CS), variable sites (VS), parsimony-informative sites (PIS), singleton sites (SS), and overall mean distance (OMD) were calculated. To assess the suitability of the ITS2 sequence as a potential barcode at the level of genus, subfamily, and family of Pinaceae, selected methods were used, namely the pairwise genetic distance-based method (PWG-distance), the DNA sequence similarity-based method (TaxonDNA, BLAST), and phylogenetic tree method (maximum likelihood).

The pairwise genetic distance-based method was used to determine the genetic divergence of the obtained ITS2 sequences. The five parameters were calculated in MEGA version X [68] using the Kimura two-parameter distance model (K2P) [69] with pairwise deletion option to define the interspecific and intraspecific variability. The interspecific divergence has been characterized by all interspecific distance and minimum interspecific distance parameters [27,70,71]. Intraspecific variability was calculated using the K2P distance matrix by applying three parameters: all intraspecific distance, theta(θ) and coalescent depth [50,70]. Using the “Pairwise summary” option based on the K2P model available in the TaxonDNA/SpeciesIdentifier 1.8 software, the frequency of the distribution of interspecific distance and intraspecific variability was obtained. Plots were made to illustrate the barcode gap for each genus and for the entire Pinaceae family.

Sequence identification and taxa discrimination rates among Pinaceae were calculated using the two different methods. The first was the method based on DNA sequence similarity-based method implemented in TAXONDNA/Species Identifier 1.8 program. “Best Match” (BM) and “Best Close Match” (BCM) options were used to verify the percentage of correct ITS2 sequence identification at the species complex, genus, subfamily and family level for taxa represented in at least two sequences [72]. The second was BLAST1. In this method, performed with the BLAST program (http://blast.ncbi.nlm.nih.gov/Blast.cgi, accessed on 15 February 2022), all ITS2 Pinaceae sequences were used as query sequences to search the reference database. Correct identification means that the best BLAST hits of the query sequence are from the expected species, while ambiguous identification means that the best BLAST hits for the query sequence turned out to be those of several species, including the expected species. Incorrect identification in turn means that the query sequence’s best BLAST hit is not from the expected species.

Phylogenetic inference was constructed by maximum likelihood (ML) analysis using 371 ITS2 Pinaceae sequences in RaxML v8.2.11 [33], with 1000 rapid bootstrap replicates along with a search for the best-scoring ML tree in every run and parsimony random seed set to 10. *Podocarpus longefoliolatus* (AY083065), *Podocarpus macrophyllus* voucher HZ20070103 (EF660588), and *Podocarpus neriifolius* isolate VNMN000814 (KR674120) were used as an outgroup.

## 5. Conclusions

Our study provides a critical assessment of nuclear DNA ITS2 region relevance for taxa discrimination among Pinaceae. Based on the results obtained, it can be concluded that although ITS2 fulfills some of the important features of an ideal DNA barcode region, its usefulness for distinguishing taxa among Pinaceae is severely limited. It seems that the correct and successful identification of taxa is reserved only for those that are phylogenetically distant and represent different genera rather than species, and for those for which few sequences are available in genetic databases. The closely related conifers at the species complex level are indistinguishable using ITS2. The application of this region for study relationships in the Pinaceae family does not seem justified due to its low genetic variability, which results low phylogenetic resolution. Nevertheless, further research on ITS2 is needed, especially in terms of extending the available genetic databases with new records, especially for species from the genera *Tsuga* or *Pseudotsuga*. A wider database would determine whether the high success in distinguishing taxa in these two genera is due to the low number of deposited samples in these databases or to the high efficiency of ITS2. Another interesting direction for further research could also be to determine the effectiveness of complete internal transcribed spacer 1 (ITS1) in discriminating Pinaceae taxa and studying the phylogeny of this large and important family of conifers.

## Figures and Tables

**Table 1:** Values of basic parameters characterizing genetic variation in ITS2 sequences in Pinaceae. AL—alignment length; CS—conserved sites; VS-variable sites; PIS—parsimony informative sites; SS—singleton sites; OMD—overall mean distance. Values in brackets are given as percentages.

Taxonomic Group/Level	Name	AL	CS	VS	PIS	SS	OMD
Species complex	*P. mugo complex*	243	243 (100.00)	0 (0.00)	0 (0.00)	0 (0.00)	0.000
Genera	*Abies*	246	169 (68.70)	75 (30.49)	27 (10.98)	48 (19.51)	0.016
	*Keteleeria*	249	118 (47.39)	122 (48.99)	0 (0.00)	121 (48.5)	0.236
	*Tsuga*	247	237 (95.95)	10 (4.05)	7 (2.83)	3 (1.21)	0.026
	*Larix*	233	172 (73.82)	61 (26.18)	15 (6.44)	46 (19.74)	0.013
	*Pseudotsuga*	232	232 (100.00)	0 (0.00)	0 (0.00)	0 (0.00)	0.000
	*Picea*	236	232 (98.31)	4 (1.69)	3 (1.27)	1 (0.42)	0.004
	*Pinus*	252	70 (27.78)	177 (70.24)	73 (28.97)	101 (40.0)	0.077
Subfamily	Abietoideae	254	66 (25.98)	186 (73.23)	88 (34.65)	97 (38.19)	0.074
	Laricoideae	233	148 (63.52)	85 (36.48)	48 (20.60)	37 (15.88)	0.023
	Pinoideae	252	61 (24.21)	187 (74.21)	107 (42.4)	77 (30.56)	0.149
Family	Pinaceae	270	17 (6.30)	250 (92.59)	196 (72.5)	49 (18.15)	0.342

**Table 2:** Values of genetic divergence indices in ITS2 sequences in Pinaceae.

Taxonomic Group/Level	Name	All Interspecific Distance	Minimum Interspecific Distance	All Intraspecific Distance	Theta	Coalescent Depth
Species complex	*P. mugo complex*	0.0000 ± 0.0000	0.0000 ± 0.0000	0.0000 ± 0.0000	0.0000 ± 0.0000	0.0000 ± 0.0000
Genera	*Abies*	0.0175 ± 0.0006	0.0000 ± 0.0000	0.0085 ± 0.0012	0.0176 ± 0.0039	0.0190 ± 0.0046
	*Keteleeria*	0.1998 ± 0.0973	0.0000 ± 0.0000	0.1972 ± 0.1919	0.1995 ± 0.0250	0.2958 ± 0.2874
	*Tsuga*	0.0356 ± 0.0119	0.0291 ± 0.0109	0.0061 ± 0.0061	0.0258 ± 00820	0.0062±0.0061
	*Larix*	0.0137 ± 0.0006	0.0000 ± 0.0000	0.0067 ± 0.0010	0.0130 ± 0.0033	0.0271 ± 0.0074
	*Pseudotsuga*	0.0000 ± 0.0000	0.0000 ± 0.0000	0.0000 ± 0.0000	0.0000 ± 0.0000	0.0000 ± 0.0000
	*Picea*	0.0048 ± 0.0009	0.0000 ± 0.0000	0.0017 ± 0.0012	0.0043 ± 0.0023	0.0024 ± 0.0018
	*Pinus*	0.0392 ± 0.0257	0.0000 ± 0.0000	0.0959 ± 0.0050	0.0768 ± 0.0102	0.0486 ± 0.0259
Subfamily	Abietoideae	0.1089±0.0082	0.0000 ± 0.0000	0.0370 ± 0.0291	0.0665 ± 0.0065	0.0562 ± 0.0389
	Laricoideae	0.0436 ± 0.0083	0.0000 ± 0.0000	0.0063 ± 0.0011	0.0222 ± 0.0037	0.0249 ± 0.0071
	Pinoideae	0.1702 ± 0.0059	0.0000 ± 0.0000	0.0342 ± 0.0229	0.1492 ± 0.0157	0.0396 ± 0.0210
Family	Pinaceae	0.3586 ± 0.0041	0.0000 ± 0.0000	0.0294 ± 0.0141	0.3417 ± 0.0287	0.0423 ± 0.0162

**Table 3:** Percentage of correct, incorrect and ambiguous sequence identifications based on the ‘best match’ and ‘best close match’ with TaxonDNA software.

Taxonomic Group/Level	Name	Best Match (BM)			Best Close Match (BCM)			
		Correct	Incorrect	Ambiguous	Correct	Incorrect	Ambiguous	No Match
Species complex	*P. mugo complex*	0.00	0.00	100.00	0.00	0.00	100.00	0.00
Genera	*Abies*	16.19	8.57	75.24	16.19	8.57	75.24	0.00
	*Keteleeria*	11.11	0.00	88.89	11.10	0.00	88.89	0.00
	*Tsuga*	100.00	0.00	0.00	100.00	0.00	0.00	0.00
	*Larix*	21.43	3.57	75.00	21.43	2.68	75.00	0.89
	*Pseudotsuga*	100.00	0.00	0.00	100.00	0.00	0.00	0.00
	*Picea*	20.83	0.00	79.17	20.83	0.00	79.17	0.00
	*Pinus*	16.22	9.01	74.77	15.32	7.21	72.07	5.40
Subfamily	Abietoideae	17.80	8.47	73.73	17.80	7.63	73.73	0.84
	Laricoideae	23.48	3.48	73.04	23.40	2.61	75.00	0.87
	Pinoideae	17.04	7.40	75.50	16.30	5.93	73.33	4.44
Family	Pinaceae	19.20	6.52	74.10	19.00	5.43	73.38	2.17

## Data Availability

All data are available within the article and Supplementary Materials.

## Supplementary Materials

The following are available online at https://www.mdpi.com/article/10.3390/plants11081078/s1, Figure S1: Evolutionary relationships analyzed 371 ITS2 sequences, Table S1: Summary of the Pinaceae samples analyzed in this study. Table S2: List of taxa and records with accession numbers analyzed in this study, Table S3: List of individuals representing *Pinus mugo* complex taxa sequenced in this study, Table S4: Abundance of all intraspecific and all interspecific K2P pairwise distance in Pinaceae, Table S5: The results of species discrimination in seven genera of the Pinaceae family based on the BLAST1 method.
