# Supplementary material for: Assessment of ITS2 Region Relevance for Taxa Discrimination and Phylogenetic Inference among Pinaceae
Source: Plants (Basel). 2022 Apr 15;11(8):1078. doi: 10.3390/plants11081078 (PMC9029031; doi:10.3390/plants11081078)
Supplement: Supplementary file 1 [file plants-11-01078-s001.zip › Table S1.pdf]

Table S1. Plant samples of Pinaceae used for analysis in this study.

Subfamily	Genus	No. of taxa	No. of sequences
Abietoideae	*Abies*	18	105
	Keteeleria	3	9
	Tsuga	2	4
Laricoideae	*Larix*	11	112
	Pseudotsuga	1	3
Pinoideae	*Picea*	7	24
	Pinus	29	111
**Total**	**7**	**71**	**368**
