# Supplementary material for: Assessment of ITS2 Region Relevance for Taxa Discrimination and Phylogenetic Inference among Pinaceae
Source: Plants (Basel). 2022 Apr 15;11(8):1078. doi: 10.3390/plants11081078 (PMC9029031; doi:10.3390/plants11081078)
Supplement: Supplementary file 1 [file plants-11-01078-s001.zip › Table S3.pdf]

Table S3. List of individuals representing *Pinus mugo* complex taxa sequenced in this study.

No.	Taxon name	Origin	Coordinates	Accession number
1	*Pinus mugo*	Tatra Mountains, Poland	49°14′30 N, 20°00′15 E	MW816475
2	*Pinus mugo*	Tatra Mountains, Poland	49°16′07 N, 20°02′41 E	MW816476
3	*Pinus uliginosa*	"Torfowisko pod Węglińcem" Nature Reserve, Poland	51°17′36 N, 15°13′37 E	MW816491
4	*Pinus uliginosa*	"Torfowisko pod Węglińcem" Nature Reserve, Poland	51°17′37 N, 15°13′34 E	MW816492
5	*Pinus* x *rhaetica*	"Bór nad Czerwonem" Nature Reserve, Poland	50° 15′42 N, 16° 8′ 31 E	MW816494
6	*Pinus* x *rhaetica*	"Bór nad Czerwonem" Nature Reserve, Poland	50° 15 42 N, 16° 8′ 31 E	MW816495
7	*Pinus uliginosa*	"Wielkie Torfowisko Batorowskie" Nature Reserve, Poland	50°27′30 N, 16°22′57 E	MW816489
8	*Pinus uliginosa*	"Wielkie Torfowisko Batorowskie" Nature Reserve, Poland	50°27′30 N, 16°22′57 E	MW816490
9	*Pinus mugo*	"Wielkie Torfowisko Batorowskie" Nature Reserve, Poland	50°49′51N, 16°20′30 E	MW816473
10	*Pinus mugo*	"Wielkie Torfowisko Batorowskie" Nature Reserve, Poland	50°50′29 N, 16°51′32 E	MW816474
11	*Pinus sylvestris*	Dendrological Garden, University of Life Sciences, Poland	52°25′32 N, 16°53′39 E	MW816487
12	*Pinus sylvestris*	Morasko, Poland	52°28′02 N, 16°55′30 E	MW816488
13	*Pinus rotundata*	Ibacher Moor, Germany	47°43′38 N, 8°03′01 E	MW816479
14	*Pinus rotundata*	Ibacher Moor, Germany	47°43′38 N, 8°03′01 E	MW816480
15	*Pinus rotundata*	Rotmeer, Germany	47°51′53 N, 8°06′00 E	MW816483
16	*Pinus rotundata*	Rotmeer, Germany	47°51′53 N, 8°06′00 E	MW816484
17	*Pinus rotundata*	Steerenmoos, Germany	47°48′21 N, 8°12′00 E	MW816485
18	*Pinus rotundata*	Steerenmoos, Germany	47°48′20 N, 8°12′00 E	MW816486
19	*Pinus rotundata*	Novohůrecká slať, Czech Republik	49°09′18 N,	MW816481

			13°19′46 E	
20	*Pinus rotundata*	Novohůrecká slať, Czech Republik	49°09′19 N, 13°19′45 E	MW816482
21	*Pinus rotundata*	Červené blato, Czech Republik	48°51′26 N, 14°48′08 E	MW816477
22	*Pinus rotundata*	Červené blato, Czech Republik	48°51′37 N, 14°48′20 E	MW816478
