# Supplementary figures and images for: Assessment of ITS2 Region Relevance for Taxa Discrimination and Phylogenetic Inference among Pinaceae
Source: Plants (Basel). 2022 Apr 15;11(8):1078. doi: 10.3390/plants11081078 (PMC9029031; doi:10.3390/plants11081078)

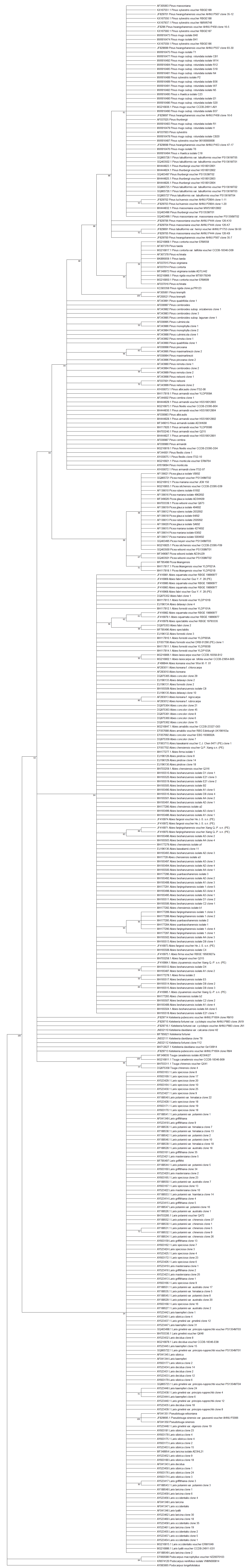

Supplement: Supplementary file 1 [file plants-11-01078-s001.zip › Figure S1.png]
